# Associations of Dietary Patterns and Risk of Hypertension in Southwest China: A Prospective Cohort Study

**DOI:** 10.3390/ijerph182312378

**Published:** 2021-11-25

**Authors:** Yixia Zhang, Yanhuan Wang, Yun Chen, Jie Zhou, Lina Xu, Kelin Xu, Na Wang, Chaowei Fu, Tao Liu

**Affiliations:** 1 Guizhou Center for Disease Control and Prevention, Guiyang 550004, China; gz_yangguang1979@126.com (Y.Z.); zhoujie19872014@163.com (J.Z.); gyvicky@163.com (L.X.); 2 School of Public Health & Key Laboratory of Public Health Safety & NHC Key Laboratory of Health Technology Assessment, Fudan University, Shanghai 200032, China; 20211020022@fudan.edu.cn (Y.W.); 18211020001@fudan.edu.cn (Y.C.); xukelin@fudan.edu.cn (K.X.); na.wang@fudan.edu.cn (N.W.)

**Keywords:** dietary patterns, factor analysis, cohort study, hypertension, China

## Abstract

Empirical data on the association between diet and incident hypertension in Southwest China is lacking. We examined the associations between various dietary patterns and the risk of incident hypertension in this prospective population cohort of Southwest China. A total of 5442 eligible adults were included from Guizhou Province, China, since 2010. Dietary information was obtained using face-to-face interviews with a semi-quantitative food frequency questionnaire, and dietary patterns were characterized using factor analysis. The hazard ratios (HRs) and 95% confidence intervals (95% CIs) were estimated for the associations between various dietary patterns and incident hypertension risk using a Cox proportional hazard model. Until 2020, a total of 1177 new hypertension cases were identified during an average follow-up of 6.97 years. In the multivariable-adjusted analysis, a low intake of the junk food pattern was significantly associated with the reducing risk of incident hypertension (HR: 0.772, 95% CI: 0.671, 0.887) and a high intake of the vegetable–grain pattern statistically lowered the risk of incident hypertension (HR: 0.774, 95% CI: 0.669, 0.894) compared with the medium intake of such patterns. Higher adherence to the vegetable–grain pattern and lower adherence to the junk food pattern significantly lowered the hypertension incidence among the population in Southwest China. Those findings suggested healthy diet guidelines should be developed for the prevention of hypertension.

## 1. Introduction

Hypertension (HTN) is a global public health issue, especially in low- and middle-income countries [1], and the prevalence has been increasing in recent decades in China [2]. The disease burden of HTN is generally complicated by possibly developing cardiovascular disease (CVD), stroke, heart failure, and kidney disease, which affect both the quality and expectancy of life [3,4]. Thus, there is a key unmet need to investigate the complex and multifaceted causes and develop more specific preventive strategies.

An imbalanced diet was highlighted as a major contributor to hypertension development [5]. However, different approaches for constructing dietary patterns and differences in their composition varied the conclusions regarding healthy patterns for HTN prevention. The Dietary Approaches to Stop Hypertension diet (DASH, characterized by a high intake of fruits, vegetables, low-fat dairy foods, and reduced saturated and total fat) was shown to be an effective dietary approach for substantially reducing blood pressure and preventing cardiovascular disease [6], and the Mediterranean dietary pattern (characterized by a high intake of fruits, vegetables, nuts, and whole grains) can decrease major clinical endpoints, including blood pressure [7]. However, since many differences exist between typical diets in Chinese and Western populations, existing diet patterns are not wholly appropriate for multiethnic Chinese with different genetic characteristics and food cultures [8].

Recently published studies paid greater attention to factor analysis [9], which takes account of the complex interactions between the different categories of dietary intake and forms dietary patterns that are targeted for local ethnic groups [10]. To our knowledge, cohort studies on specific dietary patterns in relation to the incidence risk of hypertension in China are sparse. Several cross-sectional studies have observed relationships between various dietary patterns and hypertension and called for further prospective studies [11,12,13]. Based on a cohort study of adults from Southwest China, we aimed to evaluate the associations with various dietary patterns and the risk of developing hypertension using factor analysis and to provide evidence for dietary recommendations in HTN prevention.

## 2. Materials and Methods

### 2.1. Recruitment and Study Population

The Guizhou Population Health Cohort Study (GPHCS) is an ongoing multiethnic epidemiological study of incident chronic diseases and potential risk factors in Guizhou province, China. Briefly, a total of 9280 adult residents from 48 townships of 12 districts were recruited into this prospective cohort using the multistage proportional stratified cluster sampling method. The eligibility criteria of subjects included those who (1) were aged 18 years or above, (2) lived in the study region and had no plan to move out, (3) completed survey questionnaire and blood sampling, and (4) signed the written informed consent form. Based on the intent-to-treat criteria, participants were followed up from the date of entry until death, loss to follow-up, time of a request for no further contact, or until the planned completion date in 2016–2020. During the follow-up until 2020, we updated information on the status of major chronic diseases and vital status, with a response rate of 88%. All deaths were confirmed by the unified criteria through the Death Registration Information System and Basic Public Health Service System. In this study, exclusions for participants were loss to follow-up (*n* = 1117), incomplete dietary data (*n* = 277), a history of hypertension (*n* = 2056) at baseline, and missing hypertension status (*n* = 388) during follow-up. The final analysis involved 5442 participants in this cohort (Figure S1). All participants provided written informed consent at enrollment. The study was approved by the Institutional Review Board of Guizhou Centre for Disease Control and Prevention (no. S2017-02).

### 2.2. Assessment of Dietary Patterns

Food consumption was obtained using a semi-quantitative food frequency questionnaire (FFQ) that covered 22 food and beverage items that are commonly consumed by the multiethnic residents of Southwest China at the baseline. The questionnaire was designed by the Chinese Center for Disease Control and Prevention [14] and applied in China’s chronic disease surveillance (2010). The categories included main dishes, desserts and cereals, fruits, vegetables, beverages, and dairy in this FFQ to obtain more reliable information on the dietary intake of participants in the last year. Using the food list, subjects were asked to grade their frequency of consumption of each food item using either of the four responses “times per day,” “times per week,” “times per month,” and “times per year” and their average intake per time, with the unit “50 g per day.” Food intakes were computed by multiplying the frequency of food consumption by a single intake unit of each item.

### 2.3. Assessment of Outcome and Covariates

Baseline information on socio-demographic characteristics, lifestyles, and health conditions was obtained using a structured questionnaire with a face-to-face interview. Using a standard protocol that is recommended by the World Health Organization (WHO), height and weight were measured with participants wearing lightweight clothing without shoes and recorded to the nearest 0.1 kg (or cm) [10]. The body mass index (BMI) was calculated by dividing the weight in kilograms by the square of the height in meters and categorized as recommended by the Guidelines for Prevention and Control of Overweight and Obesity in Chinese Adults [15]. All measurements were performed with quality control procedures in place. Field-measured hypertension was carried out three times per participant by trained investigators and the average of three blood pressure measurements was calculated. The Chinese Guidelines for Hypertension Prevention and Treatment (2019) were used, where hypertension was defined as an average systolic blood pressure of 140 mmHg or higher, and/or an average diastolic blood pressure of 90 mmHg or higher, and/or reporting physician-diagnosed hypertension or receiving hypertension treatment. Several variables were adjusted in the multivariable models: gender, age (<35, 35–49, ≥50 years), Han Chinese (yes, no), education years (≤9, >9 years), married (yes, no), BMI (<18.5, 18.5–23.9, 24–27.9, ≥28 kg/m^2^), farmers (yes, no), hypertension family history (yes, no), smoking (yes, no), drinking (yes, no), and regular physical activity (yes, no).

### 2.4. Statistical Analysis

Factor analysis was conducted with varimax rotation using the 16 aggregated food items, and four factors were determined according to the eigenvalue (>1), a scree plot, and the explained variation ratio. Specific food items were aggregated on the basis of the degree to which food items in the dataset were correlated with one another. Those with larger absolute factor loadings were considered to significantly contribute to the pattern. A summary score for each pattern was then derived [16] and categorized into low, medium, and high tertiles in further analysis. The factor loading matrix for the two retained dietary patterns is shown in Table S1. Factor 1, named the junk food pattern, was dominated by a high factor load of fried food, soft drinks, and desserts (0.28–0.54). The second factor was characterized by a high factor load of vegetables and grains (0.55 and 0.38) and named the vegetable–grain pattern. Common food items in the junk food pattern are partly presented in Table S2.

Person time in years was calculated for each participant from the date of enrolling the cohort to the date of diagnosis of hypertension, death, or follow-up, whichever came first. Pearson’s chi-squared test was used to compare the characteristics between subjects with different hypertension statuses at follow-up and various dietary patterns. The association between the various dietary patterns and hypertension incidence was assessed using a Cox proportional hazards regression model. The assumption of hazard proportionality in Cox regression models was tested and shown using the Schoenfeld residuals. Sensitivity analysis was conducted to investigate the potential errors and their impacts on conclusions to be drawn from the models. All statistical tests were two-sided. The data were performed using R software (Version 4.0.3; R Foundation for Statistical Computing, Vienna, Austria).

## 3. Results

### 3.1. Baseline Characteristics of Subjects

Table 1 shows the distribution of the baseline characteristics according to the follow-up hypertension status. Of the 5442 participants, three-fifths were at the age of 18–34 years and more than half were women. Newly diagnosed hypertension cases significantly tended to be older, married, farmers, having shorter education years, smokers, and fat. There were no significant differences over other variables (Table 1).

### 3.2. Distribution of Dietary Patterns

As shown in Table 2, subjects who were over 50 years old (39.2%), non-Han Chinese (41.9%), had shorter education years (40.3%), married (33.8%), farmers (39.7%), non-smokers (34.8%), non-alcohol users (34.5%), without regular physical activity (41.5%) or hypertension family history (34.7%), and normal weight (35.0%) tended to have low junk food pattern scores. Comparatively, those who were aged from 18 to 34 years (34.9%), men (37.2%), non-Han Chinese (40.3%), had longer education years (36.0%), farmers (33.6%), smokers (36.6%), and alcohol users (36.9%) tended to have high vegetable–grain food pattern scores.

### 3.3. Associations between Dietary Patterns and Hypertension Incidence

During the 37,932.5 PYs of follow-up, 1177 incident hypertension cases were documented. The junk food pattern was significantly associated with an HTN incidence risk (Table 3). Participants in the low score category of junk food pattern had a 23% lower risk of hypertension (HR: 0.783, 95% CI: 0.682, 0.899) than those in the medium intake category. After the adjustment for covariates, including lifestyles and other demographic characteristics, this association became a little stronger (HR: 0.772, 95% CI: 0.671, 0.887). Furthermore, an inverse association was observed between the vegetable–grain pattern and incident hypertension. Compared with subjects in the medium score category, those with the high scores had a lower risk of incident hypertension (HR: 0.762, 95% CI: 0.660, 0.880). Such a protective effect persisted after further adjusting these covariates (HR: 0.774, 95% CI: 0.669, 0.894).

### 3.4. Sensitivity Analysis

**Figure 1:**
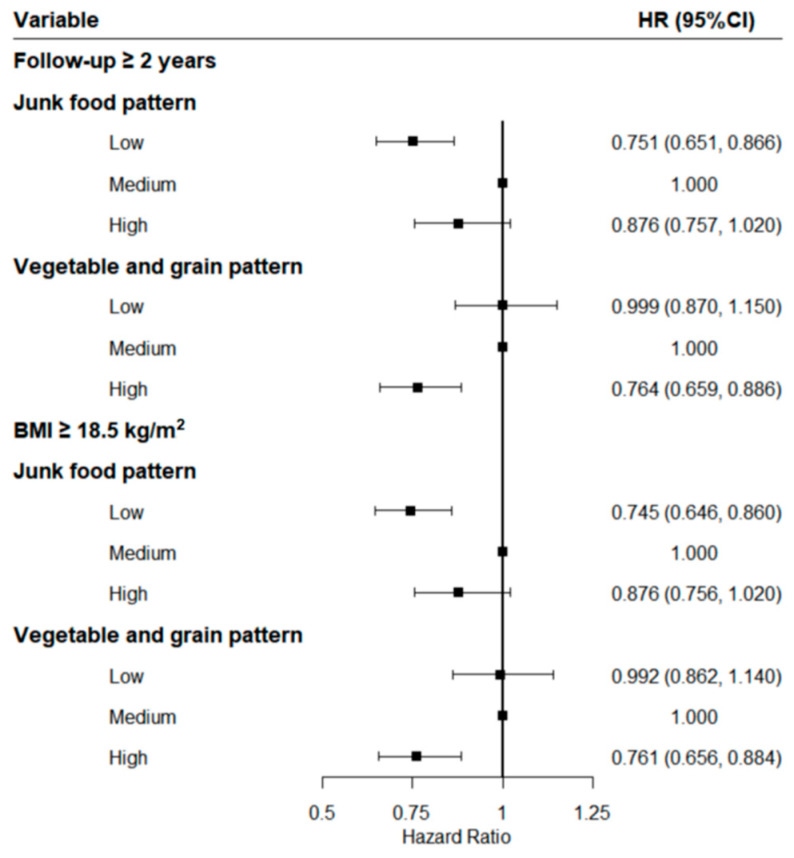
Sensitivity analysis after excluding those who entered the cohort for less than two years or were lean.

In the sensitivity analysis with participants who entered the cohort for two years or longer or those with a baseline BMI of no less than 18.5 kg/m^2^ (Figure 1), the results remained robust. Compared with the results presented above, the protective effects of the highest scores of the vegetable–grain pattern and the lowest scores of the junk food pattern did not change substantially after the adjustment for covariates.

## 4. Discussion

The risk of incident hypertension was as high as 31.03/1000 person-years among this study population in Southwest China, indicating that hypertension was an imminent health crisis for local populations. Two main dietary patterns were prominent in this community-based prospective cohort study. We found that both low adherence to the junk food pattern and high adherence to the vegetable–grain pattern were associated with significant risk reductions of incident hypertension. Those associations were independent of BMI and other HTN risk factors, and the results were virtually unchanged in the sensitivity analysis upon further restriction of participant categories. These findings suggested that offering an attractive dietary strategy may be useful in the prevention of hypertension.

Previous cohort studies or meta-analyses demonstrated that the DASH diet reduces blood pressure and is recommended for preventing cardiovascular disease [6,17]. The DASH diet emphasizes the intake of fruits, vegetables, low-fat dairy foods, and reduced saturated and total fat. However, Southwest China is a multi-minority community with different genetic characteristics [18], and different ethnic groups have specialized food preferences and food cultures for geographical and economic reasons [19]. The habit that features beef, mutton, and pickles as the main component still exists among residents. Vegetables are commonly eaten steamed or boiled and mixed with seasonings rather than eaten raw. Therefore, the DASH is not very appropriate for evaluating the dietary quality of people in China. Factor analysis was conducted in this study to characterize dietary patterns based on the main food types that are consumed locally and evaluate the dietary quality among the population in Southwest China.

In China, the traditional diet greatly changed from low to high in fat and energy density with the rapid growth of the economy [20], and this remarkable nutrition transition may be a better explanation of the high rates of diet-related non-communicable disease in the past few decades [21]. In the current analysis, low adherence to the junk food pattern was associated with a 23% lower risk of incident HTN compared with the medium category after an adjustment for potential covariates. The underlying biological mechanisms may be explained as a high-fat-diet-induced sensitization of angiotensin-II-elicited hypertension is mediated by leptin through the upregulation of the central renin–angiotensin system and proinflammatory cytokines [22]. The association between avoiding excessive consumption of carbohydrates and sodium and promoted prevention of hypertension was further supported by a study based on 14,338 South Korean women [23]. Moreover, high saturated fat and sugar intake was found to be associated with accelerated development of cardiac pathology and dysfunction in hypertensive subjects [24,25]. However, the increased risk was not observed to be significant in the high level of such a pattern. One possible reason was that the intake did not reach the dangerous thresholds in the study population. Another was that the sample size was not enough for such an association in this study.

Moreover, it was observed that high adherence to the vegetable–grain pattern was associated with a 23% decreased risk of HTN, which was consistent with previous studies [5,26]. A 6-year follow-up study based on the Korean Genome and Epidemiology Study cohort demonstrated that women in the highest tertile of the whole grains and legumes pattern scores had a lower risk of incident hypertension [5]. Similar dietary patterns were reported by a randomized clinical trial involving Swedish women [26]. Grains and vegetables contain various nutrients and food components, which contribute to a synergistic and favorable effect on hypertension [27,28]. Vitamins and minerals that are included in vegetables and dietary fiber in whole grains have antihypertensive effects [29], and magnesium in diets directly moderates blood pressure by controlling the contraction or vasodilation of vascular cells [30]. Furthermore, a higher intake of whole grains and legumes was inversely related to specific cellular adhesion molecules and the reactive hyperemia index, which serve as surrogates of endothelial dysfunction and peripheral vascular function, respectively [31,32]. In addition, the components of whole grains and legumes may induce an increase in insulin sensitivity and anti-inflammatory markers, further lowering blood pressure levels [26,33].

Nevertheless, the finding remains controversial. No association was observed between vegetable consumption and risk of incident hypertension in middle-aged and older Korean adults [34]. This pattern only shared vegetables of food items in the vegetable-grain pattern, but no grain category was included. This may partly be attributed to the fact that a combination of vitamins, minerals, and bio-active compounds contributes to a lower hypertension risk than the intake of a single nutrient alone. Moreover, different effects in various studies may be caused by the inconsistency of study designs, demography characteristics, duration of follow-up time, and methods of measurement. In this study, the per capita daily intake of vegetables (355.35 g) in the second tertile of the vegetable–grain pattern met the amount (300–500 g) that is recommended by the Dietary Guidelines for Chinese Residents (2016 Edition) [35] and, therefore, was used as the reference group.

Except for a high-salt diet as a well-established detrimental factor for hypertension, divergent effects among different food groups on hypertension risk remain unclear. Furthermore, evidence from cohort studies that focus on the effects of various dietary patterns on incident hypertension in Southwest China is lacking. The strengths of this study should be noted. First, the longitudinal study and long duration assisted in making causal inferences between dietary patterns and new-onset hypertension. Second, the analysis of food consumption in the form of dietary patterns offered a comprehensive approach to disease prevention by addressing the collective health benefit of the whole diet and enhancing the applicability and sustainability in practice. Several diet patterns that were unique to the local customs were identified using factor analysis. Further strengths also included the genetic and socioeconomic variation with a considerable proportion of Chinese minorities, as well as a high follow-up rate (88%). Of course, some limitations should be discussed. One of the important limitations of this study was the possibility of measurement error from the self-reported diet assessment. Despite the adjustment for potential confounders, residual confounding cannot be ruled out. Moreover, the subjectivity in factor analysis modeling might have slight impacts on the results, but the dietary patterns were generally consistent in this cohort and the findings remained robust in the subgroups. In addition, diet may change during the 7-year follow-up period but we could not evaluate such change in those diet patterns and its possible impact on the risk of incident hypertension.

## 5. Conclusions

In conclusion, the junk food pattern was associated with a higher risk of incident hypertension and the vegetable–grain pattern was associated with a lower risk of incident hypertension in Southwest China. The diverse cohort offered some insights into the etiology of the association between dietary patterns and incident hypertension, and these findings provide additional support for dietary intervention strategies to improve population diet patterns, such as shifting from a junk food pattern to a vegetable–grain pattern in order to combat the growing disease burden among multi-ethnic Chinese populations.

## Figures and Tables

**Table 1:** Baseline characteristics among the community population in Southwest China.

Characteristics	Total	Non-HTN	New HTN	*p*-Value
Participants, n	5442	4265	1177	
Age, years				<0.001
18.0–34.9	3267 (60.0)	2739 (64.2)	528 (44.9)	
35.0–49.9	1542 (28.3)	1119 (26.2)	423 (35.9)	
≥50.0	633 (11.6)	407 (9.5)	226 (19.2)	
Women, %	2970 (54.6)	2357 (55.3)	613 (52.1)	0.056
Han Chinese, %	3096 (56.9)	2397 (56.2)	699 (59.4)	0.055
Education > 9 years, %	2453 (45.1)	2020 (47.4)	433 (36.8)	<0.001
Married, %	4380 (80.5)	3404 (79.8)	976 (82.9)	0.019
Farmer, %	3111 (57.2)	2373 (55.6)	738 (62.7)	<0.001
Current smoker, %	1484 (27.3)	1128 (26.4)	356 (30.2)	0.011
Alcohol use, %	1691 (31.1)	1324 (31.0)	367 (31.2)	0.956
Physical activity, %	4717 (86.7)	3695 (86.6)	1022 (86.8)	0.899
HTN family history, %	522 (9.6)	421 (9.9)	101 (8.6)	0.203
BMI, kg/m^2^				0.001
<22.0	357 (6.6)	290 (6.8)	67 (5.7)	
22.0–23.9	3585 (66.0)	2844 (66.8)	741 (63.1)	
24.0–27.9	1233 (22.7)	942 (22.1)	291 (24.8)	
≥28.0	257 (4.7)	181 (4.3)	76 (6.5)	

Abbreviations: HTN, hypertension; BMI, body mass index.

**Table 2:** Tertiles of dietary patterns over subjects with different baseline characteristics in Southwest China (%).

Characteristics	Junk Food Pattern				Vegetable–Grain Pattern			
	Low	Medium	High	*p*-Value	Low	Medium	High	*p*-Value
Participants, n	1814	1814	1814		1814	1814	1814	
Age, years				<0.001				<0.001
18.0–34.9	1013 (31.0)	1032 (31.6)	1222 (37.4)		985 (30.1)	1141 (34.9)	1141 (34.9)	
35.0–49.9	553 (35.9)	551 (35.7)	438 (28.4)		537 (34.8)	500 (32.4)	505 (32.7)	
≥50.0	248 (39.2)	231 (36.5)	154 (24.3)		292 (46.1)	173 (27.3)	168 (26.5)	
Gender				0.101				<0.001
Women	953 (32.1)	1011 (34.0)	1006 (33.9)		1096 (36.9)	980 (33.0)	894 (30.1)	
Men	861 (34.8)	803 (32.5)	808 (32.7)		718 (29.0)	834 (33.7)	920 (37.2)	
Han Chinese				<0.001				<0.001
Yes	832 (26.9)	982 (31.7)	1282 (41.4)		1206 (39.0)	1021 (33.0)	869 (28.1)	
No	982 (41.9)	832 (35.5)	532 (22.7)		608 (25.9)	793 (33.8)	945 (40.3)	
Education years				<0.001				0.001
>9	608 (24.8)	729 (29.7)	1116 (45.5)		772 (31.5)	799 (32.6)	882 (36.0)	
≤9	1206 (40.3)	1085 (36.3)	698 (23.4)		1042 (34.9)	1015 (34.0)	932 (31.2)	
Married				<0.001				0.677
Yes	1480 (33.8)	1512 (34.5)	1388 (31.7)		1449 (33.1)	1470 (33.6)	1461 (33.4)	
No	334 (31.5)	302 (28.4)	426 (40.1)		365 (34.4)	344 (32.4)	353 (33.2)	
Farmers				<0.001				<0.001
Yes	1236 (39.7)	1082 (34.8)	793 (25.5)		974 (31.3)	1093 (35.1)	1044 (33.6)	
No	578 (24.8)	732 (31.4)	1021 (43.8)		840 (36.0)	721 (30.9)	770 (33.0)	
Current smoker				<0.001				<0.001
Yes	437 (29.4)	493 (33.2)	554 (37.3)		439 (29.6)	502 (33.8)	543 (36.6)	
No	1377 (34.8)	1321 (33.4)	1260 (31.8)		1375 (34.7)	1312 (33.1)	1271 (32.1)	
Alcohol use				<0.001				<0.001
Yes	519 (30.7)	530 (31.3)	642 (38.0)		472 (27.9)	595 (35.2)	624 (36.9)	
No	1295 (34.5)	1284 (34.2)	1172 (31.2)		1342 (35.8)	1219 (32.5)	1190 (31.7)	
Physical activity				<0.001				0.267
Yes	1513 (32.1)	1552 (32.9)	1652 (35.0)		1570 (33.3)	1557 (33.0)	1590 (33.7)	
No	301 (41.5)	262 (36.1)	162 (22.3)		244 (33.7)	257 (35.4)	224 (30.9)	
HTN family history, %				<0.001				0.629
Yes	107 (20.5)	158 (30.3)	257 (49.2)		166 (31.8)	183 (35.1)	173 (33.1)	
No	1707 (34.7)	1656 (33.7)	1557 (31.6)		1648 (33.5)	1631 (33.2)	1641 (33.4)	
BMI, kg/m^2^				0.013				0.444
<22.0	117 (32.8)	124 (34.7)	116 (32.5)		123 (34.5)	134 (37.5)	100 (28.0)	
22.0–23.9	1254 (35.0)	1169 (32.6)	1162 (32.4)		1190 (33.2)	1181 (32.9)	1214 (33.9)	
24.0–27.9	370 (30.0)	428 (34.7)	435 (35.3)		410 (33.3)	414 (33.6)	409 (33.2)	
≥28.0	70 (27.2)	87 (33.9)	100 (38.9)		87 (33.9)	82 (31.9)	88 (34.2)	

Abbreviations: HTN, hypertension; BMI, body mass index.

**Table 3:** The incidence risk of hypertension according to tertiles of dietary patterns among participants in Southwest China.

	Cases, *n*	Incident Density/1000 PYs	HR (95% CI)		
			Model 1	Model 2	Model 3
Junk food pattern					
Low	376	29.52	0.783 (0.682, 0.899) ***	0.771 (0.671, 0.886) ***	0.772 (0.671, 0.887) ***
Medium	435	34.60	1.000	1.000	1.000
High	366	34.00	0.865 (0.752, 0.994) *	0.917 (0.794, 1.060)	0.895 (0.775, 1.030)
Vegetable and grain pattern					
Low	416	33.11	0.964 (0.842, 1.100)	0.986 (0.860, 1.130)	0.990 (0.864, 1.140)
Medium	438	34.26	1.000	1.000	1.000
High	323	25.67	0.762 (0.660, 0.880) ***	0.775 (0.670, 0.895) ***	0.774 (0.669, 0.894) ***

Note: Model 1—adjusted for age, gender; model 2—model 1 plus nationality, education, marriage, occupation, hypertension family history, and baseline BMI value; model 3—model 2 plus smoking status, alcohol use, and regular physical exercise. ***: *p* < 0.001, *: *p* < 0.05. Abbreviations: PY, person-years; HR, hazard ratio; 95% CI, 95% confidence interval.

## Data Availability

The datasets that were generated for this study are available on request to the corresponding author.

## Supplementary Materials

The following are available online at https://www.mdpi.com/article/10.3390/ijerph182312378/s1, Figure S1: The flow chart, Table S1: Factor loading matrix after the dietary pattern rotation and average daily intake of each food group (g), Table S2: Common food list of the junk food pattern.
