# Supplementary material for: Associations of Dietary Patterns and Risk of Hypertension in Southwest China: A Prospective Cohort Study
Source: Int J Environ Res Public Health. 2021 Nov 25;18(23):12378. doi: 10.3390/ijerph182312378 (PMC8656527; doi:10.3390/ijerph182312378)
Supplement: Supplementary file 1 [file ijerph-18-12378-s001.zip › ijerph-1465891.pdf]

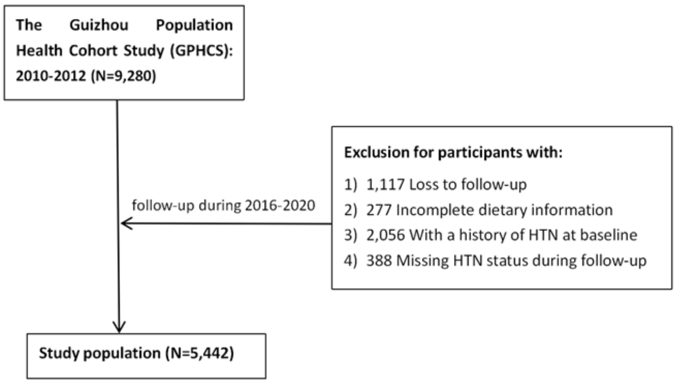

Figure S1 The flow chart

Abbreviations: HTN, hypertension.

Table S1 Factor loading matrix after dietary pattern rotation and average daily intake of each food group (g)

**Food category**	**Junk food pattern**				**Vegetable-grain pattern**			
	Low	Medium	High	Factor loading	Low	Medium	High	Factor loading
Grain (rice, flour and coarse cereals, etc)	499.34	381.06	384.76	-0.07	296.31	405.73	563.12	**0.38**
Potato (potato, taro and sweet potato, etc)	11.13	20.23	35.83	0.14	21.54	19.09	26.54	0.05
Meat (pork, beef, mutton, chicken, duck, etc)	78.22	100.73	133.39	0.12	60.78	87.85	163.71	0.32
Fish (fish, shrimp, crab, etc)	3.39	6.18	17.08	0.09	4.65	7.86	14.15	0.12
Vegetable (light- and dark-colored vegetables)	448,42	351.28	365.32	-0.04	193.11	355.35	616.56	**0.55**
Fruit (apple, banana, etc)	27.43	45.78	101.60	0.15	32.51	48.25	94.06	0.26
Egg	15.52	11.15	20.85	0.07	18.43	14.15	14.93	-0.05
Milk	1.94	4.44	33.17	0.16	15.51	11.10	12.94	-0.01
Legume (soybean, mung,	29.38	17.48	31.34	0.04	21.67	24.35	32.18	0.08

etc)								
Friedfood (seedcake, deep-fried dough stick, etc)	0.37	1.72	11.49	**0.28**	4.28	4.17	5.12	0.00
Soft drink (fruit beverage, carbonated drinks, etc)	0.77	4.86	76.63	**0.54**	26.21	30.15	25.90	-0.01
Dessert (bread, cake, etc)	0.26	1.06	14.23	**0.51**	6.78	4.76	4.00	-0.05
Oil (soybean oil, olive oil, etc)	32.29	47.83	54.17	0.10	43.81	46.43	44.06	-0.01
Pickles	7.83	4.94	6.58	-0.02	5.52	5.14	8.69	0.07
Sufu	2.01	0.95	1.69	0.00	2.07	1.06	1.51	-0.03
Soybean paste	1.38	0.75	1.77	0.02	1.81	0.88	1.21	-0.04

Table S2 Common food list of the junk food pattern

**Food category**	**Food item**	**Local name**
Friedfood	Seedcake	“油饼”
	Deep-fried dough stick	“油条”
Soft drink	Cola	“可乐”
	Sprite	“雪碧”
Dessert	Pineapple cake	“凤梨酥”
	Yolk pie	“蛋黄派”
	Mooncake	“月饼”
