# Supplementary figures and images for: Associations of Dietary Patterns and Risk of Hypertension in Southwest China: A Prospective Cohort Study
Source: Int J Environ Res Public Health. 2021 Nov 25;18(23):12378. doi: 10.3390/ijerph182312378 (PMC8656527; doi:10.3390/ijerph182312378)

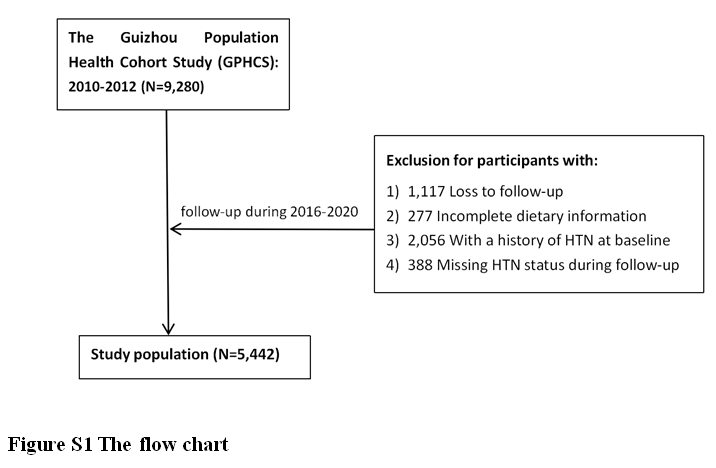

Supplement: Supplementary file 1 [file ijerph-18-12378-s001.zip › Figure S1 (with title).tif]

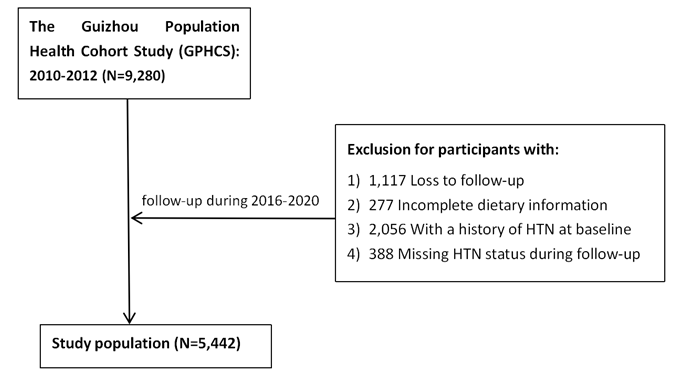

Supplement: Supplementary file 1 [file ijerph-18-12378-s001.zip › Figure S1.tif]
